# Concentration Changes of Peripheral Blood Mesenchymal Stem Cells of Sprague Dawley Rats during Distraction Osteogenesis

**DOI:** 10.1111/os.12823

**Published:** 2021-02-09

**Authors:** Gang‐qiang Du, Zhi‐hao Gong, Bin Liang, Peng Li, Shu‐ye Yang, Long Jia, Jian‐hao Jiang, Kai Zhang

**Affiliations:** ^1^ Department of Orthopaedic Trauma Binzhou Medical University Hospital Binzhou China; ^2^ Department of Orthopaedic Surgery The People's Hospital of Zhao Yuan City Yantai China

**Keywords:** Distraction osteogenesis, Mesenchymal stem cells, Peripheral blood, Sprague–Dawley rats

## Abstract

**Objectives:**

To observe the changes in the concentrations of circulating peripheral blood mesenchymal stem cells (PBMSCs) in Sprague Dawley (SD) rats and explore the pattern of changes in PBMSCs during the process of distraction osteogenesis.

**Methods:**

SD rats were randomly divided into the osteotomy with lengthening group (lengthening group), the osteotomy without lengthening group (osteotomy group), and the blank control group (control group). Each group included 24 rats. Percutaneous pinning with external fixation of the left femur was carried out in lengthening group and osteotomy group, but control group received no surgical treatment. On day 5 after operation, continuous traction was carried out at a rate of 0.25 mm/d in lengthening group, while no traction was carried out in osteotomy group. Peripheral blood was collected from all rats on days 1, 3, 7, and 16 after the start of traction. PBMSCs were isolated by density gradient centrifugation. CD105, CD34, and CD45 were selected as cell surface markers. The concentration of PBMSCs was detected by flow cytometry and compared between groups at different time points. X‐ray films were taken during and after the operation to observe whether the osteotomy end was pulled and the growth and mineralization of the new bone in the osteogenic area of the femur. Color ultrasound was used to monitor the width of the distraction space, the formation of new bone, and the blood supply of soft tissue around the distraction.

**Results:**

All rats were able to tolerate the operation well, and the external fixation was firm and reliable. X‐ray showed that, in lengthening group, the distraction space of femur gradually widened and new bone gradually formed in the distraction space; after 8 weeks, the samples were taken out, which showed that the new bone tissue in the lengthened area healed well. In osteotomy group, the average healing time of osteotomy was (7.12 ± 0.78) weeks. Ultrasonic examination showed that after the end of traction, the high echo callus shadow was seen in the traction space, and the blood flow signal was obviously rich at an earlier stage. In lengthening group and osteotomy group, the average concentrations of PBMSCs (3.02% ± 0.87% *vs* 2.95% ± 0.74%, respectively) were significantly increased in the early stage after osteotomy, and the average concentrations of PBMSCs on days 3, 7, and 16 after the start of traction were 5.34% ± 1.13% *vs* 3.28% ± 1.22%; 6.41% ± 1.05% *vs* 3.16% ± 0.92%; and 5.94% ± 1.23% *vs* 1.48% ± 0.52%, respectively. The concentration of PBMSCs in peripheral blood of lengthening group and osteotomy group was the same at osteotomy stage, and the difference between the two groups was not statistically significant (*P* > 0.05). After that, compared with lengthening group, the concentration of PBMSCs in osteotomy group gradually decreased and maintained at a certain level; the difference between the two groups was statistically significant (*P* < 0.05).

**Conclusions:**

Distraction osteogenesis of femur can significantly increase PBMSCs in SD rats and participate in the process of bone formation.

## Graphical abstract

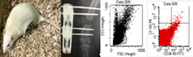

In the rat model of femoral osteotomy, with the process of distraction osteogenesis, peripheral blood mesenchymal stem cells will have obvious aggregation changes.

## Introduction

The technique of distraction osteogenesis applies the law of tension‐stress (LTS) by cutting a bone with blood supply and applying a movable device to the external fixator at a specific rate and frequency to promote formation of new bone tissue in the traction gap, and thus achieve limb lengthening and deformity correction^1^, ^2^. Distraction osteogenesis is a complicated cascade reaction involving multiple cells and cytokines, with tissue regeneration and differentiation occurring throughout the whole process. Numerous studies have shown that mesenchymal stem cells (MSCs) are concentrated in the reconstructed tissues during tissue regeneration and reconstruction^3^, ^4^, ^5^.

Distraction osteogenesis is a complex process involving many kinds of cells and cytokines. When mechanical stress stimulation acts on bone tissue, mesenchymal stem cells, osteoblasts, and osteoclasts are all involved in the process of bone absorption and bone regeneration, mainly including the proliferation and differentiation of various cells, the formation of new blood vessels, and the mineralization and reconstruction of new bone tissue^6^. The process of bone regeneration is closely related to the changes of cell morphology and function. Sailhan *et al*.^7^ believed that under the stimulation of mechanical stress, the ultrastructures of cells would change, such as mitochondria and endoplasmic reticulum, Golgi would be hypertrophic, and the number of intracellular vesicles would increase. The cells are in active anabolism, the energy supply of cells is increased, and many kinds of proteins are synthesized vigorously. The migration of mesenchymal stem cells to the tractive space is mainly from bone marrow, periosteum and peripheral blood. Under the mechanical stress stimulation, the mesenchymal stem cells that migrated to the distraction gap can be differentiated into bone, cartilage, muscle, bone marrow matrix, fat, and other tissues to fill the bone defect caused by distraction^8^. Mobilization and migration of mesenchymal stem cells into the distraction gap are considered to be the key steps to determine the treatment cycle of distraction osteogenesis^9^. However, how mechanical stress stimulation can be converted into biochemical signals in distraction osteogenesis needs further study.

Adequate blood supply is needed for metabolism of new bone tissue during distraction. Jiang *et al*.^9^ found that in the process of distraction osteogenesis, the blood flow in the distraction gap was more abundant than that in the callus during fracture healing. Mechanical stress stimulation can promote the formation of new blood vessels, which has been confirmed by a large number of studies, and can also increase the local blood flow of the affected limb. It was found that the expression of genes related to angiogenesis increased at the beginning of the stretch phase, which confirmed that mechanical stress stimulation can promote angiogenesis. At the same time, it was found that the number and density of new blood vessels in low‐speed traction were much higher than that in high‐speed traction. During osteotomy, the blood circulation of the original bone segment will be destroyed, resulting in local hypoxia, and the reduction of oxygen tension starts the angiogenesis process^6^. The angiogenesis in the distraction space is prior to the formation of bone tissue. The formation of new blood vessels and the increase of blood flow provide sufficient blood supply for the metabolism of new bone tissue.

Mesenchymal stem cells have a wide range of sources and can be isolated from various tissues, such as bone marrow, adipose tissue, cord blood, and peripheral blood. The traditional view holds that it is an ideal choice to obtain mesenchymal stem cells from bone marrow and adipose tissue, but the acquisition from bone marrow and adipose tissue is a painful and invasive process^10^. Peripheral blood extraction process is simple, the damage to human body is small, and there is no late complications. Therefore, circulating peripheral blood is an ideal choice to obtain mesenchymal stem cells^11^. There are many kinds of adult stem cells in the circulating peripheral blood, such as hematopoietic stem cells, endothelial progenitor cells, mesenchymal stem cells, and bone progenitor cells^12^. Zheng *et al*.^11^, ^13^ found that bone marrow‐derived mesenchymal stem cells and peripheral blood‐derived mesenchymal stem cells can differentiate into osteoblast‐related cells, and have similar biological characteristics in inhibiting cell apoptosis.

**Fig. 1:**
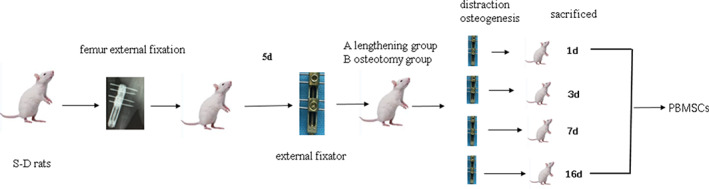
Distraction osteogenesis model. SD rats were randomly divided into the osteotomy with lengthening group (lengthening group), the osteotomy without lengthening group (osteotomy group). Percutaneous pinning with external fixation of the left femur was carried out in each group. On day 5 after operation, continuous traction was carried out in lengthening group, while no traction was carried out in osteotomy group. Peripheral blood was collected from all rats on days 1, 3, 7, and 16 after the start of traction. PBMSCs were isolated by density gradient centrifugation. CD105, CD34, and CD45 were selected as cell surface markers. The concentration of PBMSCs was detected by flow cytometry and compared between groups at different time points.

We speculate that the concentration of peripheral blood mesenchymal stem cells (PBMSCs) changes with the traction during the process of distraction osteogenesis, but no relevant studies have been conducted. On this basis, we established a distraction osteogenesis model in the femur of Sprague Dawley (SD) rats to observe the change in the concentration of PBMSCs in order to i) explore whether the body mobilizes and recruits MSCs to the distraction gap to “help” osteogenesis, and ii) provide a theoretical basis for consideringMSCs as a therapeutic target for poor bone formation, and iii) examine excessively long treatment during distraction osteogenesis (distraction osteogenesis model, Fig. 1).

## Materials and Methods

### *Establishment of Distraction Osteogenesis Model in the Femur of* *SD* *Rats*

This study followed the “Principles of Laboratory Animal Care” and was approved by the Animal Ethics Committee of Binzhou Medical University. We obtained 8‐week‐old male specific‐pathogen‐free SD rats (supplied by Jinan Pengyue Experimental Animal Breeding Co., Ltd., Jinan, China) weighing 360–390 g. After 1 week of adaptive feeding in the laboratory, 72 rats with normal diet were included in the experiment. These rats were randomly divided into three equal groups.

For the rats in lengthening group and osteotomy group, osteotomy was performed first. The procedure was as follows. Anesthesia was given using intraperitoneal injection of 10% chloral hydrate (0.3 mL/100 g). Parallel threaded pins with a diameter of 1.2 mm were inserted perpendicular to the centerline of the femur from sites 5, 10, 20, and 25 mm distal to the farthest end of the lateral femoral condyle, penetrating both cortexes and the opposite side skin surface. An anterolateral approach to the femoral shaft was used. A longitudinal incision about 10 mm long was made in the skin surface above the intermuscular space between the rectus femoris and the vastus lateralis. The rectus femoris and vastus lateralis were bluntly separated to expose the femoral shaft. The peripheral soft tissue and periosteum were protected using hemostatic clamps. A Kirschner wire 0.8 mm in diameter was used to make several holes at the osteotomy line, and a pair of surgical scissors were used to cut the femur along the osteotomy line. The external fixator was installed. A wrench matching this micro external fixator was used to rotate the nuts several times to confirm that the osteotomy was complete. The nuts were adjusted to compress the broken end of the osteotomy, and each clasp was locked with maximum torque. During the operation, X‐ray films were taken to determine that the osteotomy was complete; to check for a fracture at the pin tract; and to confirm the alignment of the two bone segments after the osteotomy, allowing for timely adjustment of the frame of the external fixator. After the surgical procedure was completed, repeated irrigation was carried out using a large amount of normal saline. The incision was sutured layer by layer using a 3‐0 silk thread and wrapped with sterile gauze. Nothing was done to the control group.

### *Postoperative Management*

For the animals undergoing surgery, the surgical incision was observed daily after surgery and penicillin (100,000 units/d) was administered intramuscularly for 3 days after the osteotomy. The suture was removed about 10 days after surgery based on the healing of the incision. The skin around the site of the pin insertion was sterilized using alcohol to prevent pin site infection. Each experimental rat was fed in a single cage after operation, and observed for loosening of the external fixation bolt, slipping of the threaded pin, and infection in the skin incision or at the pin site.

**Fig. 2:**
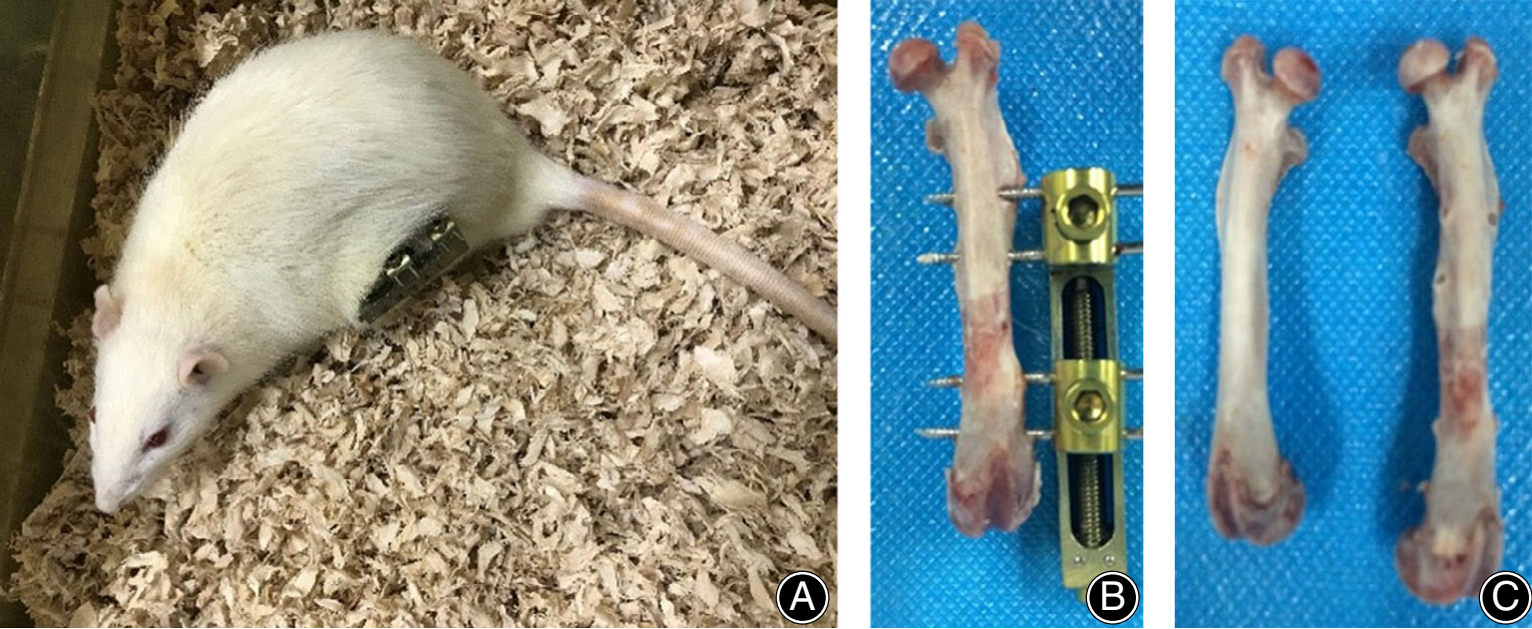
Postoperative appearance of Sprague Dawley rats. (A) SD rats can move freely after osteotomy. (B) Rotate the nut in the tractor, rotate the bone for one turn to extend to 0.25 mm, and our speed is extended to 0.25 mm/d. After 8 weeks of mineralization, the femur was dissected and the new bone tissue was close to the normal bone tissue. (C) Shows that the extension rate of the left extended femur is about (5.12 ± 0.73)% compared with the opposite.

The postoperative management of external fixation in the various groups was as follows. In lengthening group, after 5 days of rest, limb lengthening was carried out for a total of 16 days at the rate of 0.25 mm/d (rotate the nut in the tractor for one turn, and extend the external fixation to 0.25 mm, Fig. 2B), and then stopped. In osteotomy group, no limb lengthening was carried out after percutaneous pinning and external fixation. In control group, no surgery was performed. In each group, peripheral blood was obtained on post‐operation days 1, 3, 7, and 16. The MSCs were separated by density gradient centrifugation. CD105, CD34, and CD45 were selected as the surface markers of MSCs, and samples were detected by flow cytometry. The blood samples were taken and analyzed at the same time points in the lengthening group and the osteotomy group.

### *Separation of* *PBMSCs* *Using Density Gradient Centrifugation*

Six animals were sacrificed in each group on day 1, 3, 7, and 16 of the distraction period, and 4 mL of blood was taken from the heart of each rat and treated by heparin anticoagulation. The sample was diluted with the same amount of sample diluent. To a 15 mL centrifuge tube was added equal amounts of the separating medium and the diluted sample. The diluted blood sample was carefully taken by a plastic pipette and added on the surface of the separating medium. The mixed samples were placed in a tabletop high‐speed centrifuge with a centrifugal force of 560 gat at 20 °C for 30 min.

### *Analysis of Samples Using Flow Cytometry*

CD105, CD34, and CD45 were selected as the surface markers of MSCs. Briefly, 500 μL of washed samples were resuspended and transferred into a flow tube. CD105‐PE, CD45‐FITC, and CD34‐FITC were sequentially added to each sample at 4 °C and incubated in a 4 °C refrigerator for 50 min. After incubation, the samples were analyzed using flow cytometry.

### *Monitoring with X‐ray and Ultrasonography*

X‐ray films were taken during and after surgery to observe the state of distraction in the osteotomy ends, the alignment of the two bone segments, any fractures at the pin site, and the growth mineralization of the new bone in the femoral osteogenesis area. The unified X‐ray film shooting standard was: mobile X‐ray machine, voltage 42 kV, current 100 mA, focal length 80 cm, and exposure time 0.08 s.

The Logiq E9 color ultrasound instrument (GE Healthcare, Chicago, IL) was used with a probe frequency of 4 to 9 MHz. The intraoperative and postoperative width of the distraction gap, the formation of new bone, and the blood supply of the soft tissue around the distraction gap were monitored. We recorded any slight linear echo in the gap, the formation of any new blood vessels, and blood flow change in the gap. In addition, the blood supply of the soft tissue around the osteogenesis was observed.

### *Statistical Analysis*

The data obtained from the experiment were put into the computer, and the data were statistically processed using the Statistical Product and Service Solutions (SPSS) 19.0 software (IBM Corp., Armonk, NY). The experimental data were expressed as mean ± standard deviation. The sample data of various groups were compared by one‐way analysis of variance (ANOVA). *P* < 0.05 was considered statistically significant.

## Results

### *General Information*

All rats successfully survived the perioperative period, and none died during the experiment. The rats had poor mental state, diet, and free movement ability in the first 2 days after surgery, but recovered to the normal preoperative level on day 3 after surgery (Fig. 2).

### *Imaging Examination*

**Fig. 3:**
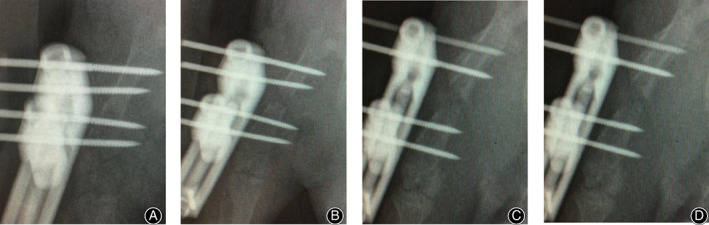
X‐ray monitoring during femoral distraction osteogenesis in Sprague Dawley rats. (A) X‐ray film taken after osteotomy showed that the alignment of the two bone segments in the middle part of the left femur was excellent. (B–D) X‐ray films taken on days 1 (B), 7 (C), and 16 (D) after distraction showed that the distraction gap became wider, the osteotomy line was obvious, and the alignment of the two bone segments was excellent.

X‐ray examination of the rats in the experimental group was performed at different time points of the distraction period. Along with the lengthening, the distraction gap was gradually widened, the alignment of the two bone segments became excellent, and there was a translucent image in the gap at the end of the distraction (Fig. 3).

**Fig. 4:**
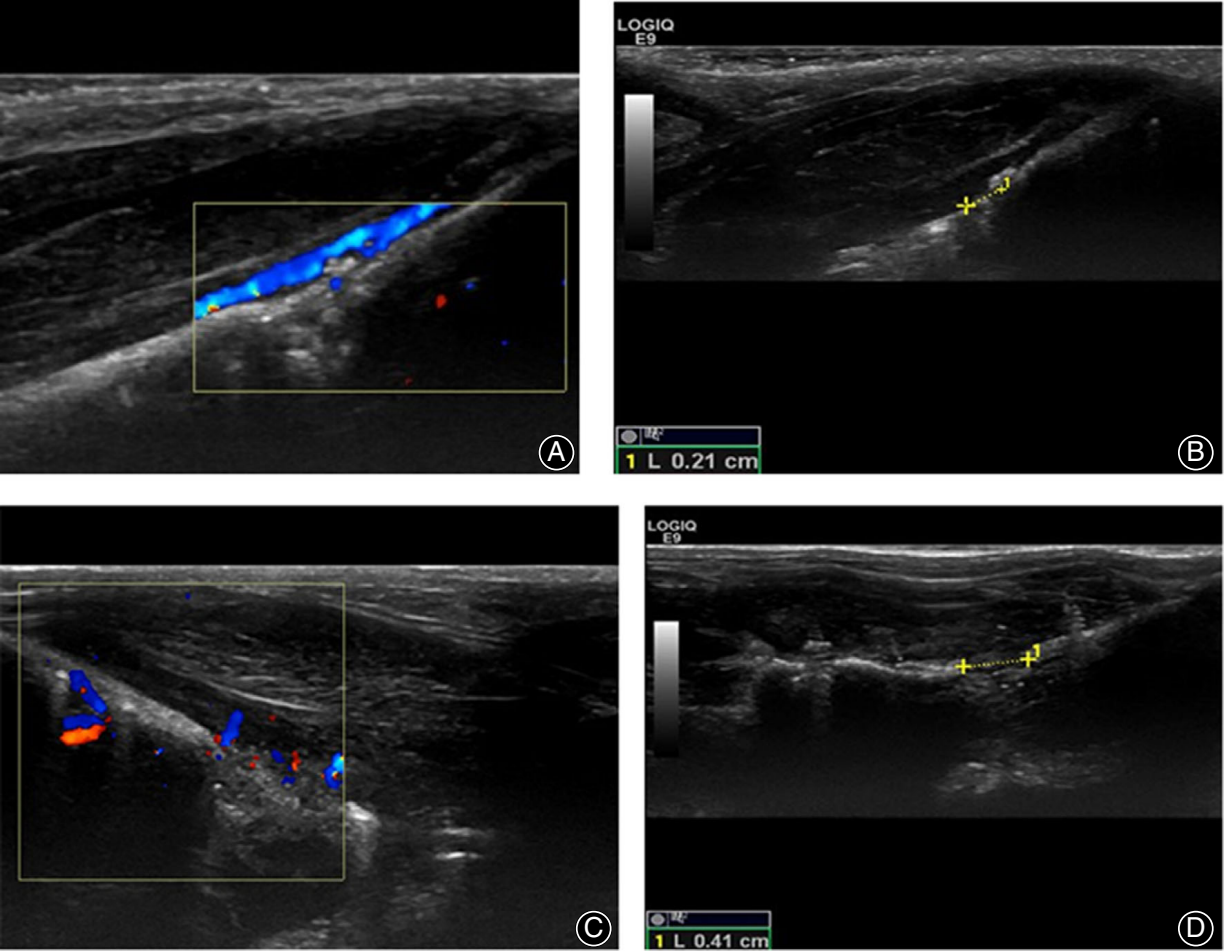
Ultrasonography monitoring during femoral distraction osteogenesis in Sprague Dawley rats. (A) On day 8 after distraction, spotty blood flow signals were observed in the distraction gap. (B) On day 8 after distraction, the width of the distraction gap was measured and found to be close to the expected length. (C) At the end of distraction, neovascularization was observed in the distraction gap. (D) At the end of distraction, the width of the distraction gap was measured and found to be close to the expected length.

Ultrasonography was performed on the left lower extremity of the rats after operation to observe whether there was a stable blood flow from the left femoral artery to the osteotomy end. No femoral artery injury occurred during surgery. The distraction gap was measured on day 8 after the start of distraction, and the widths of all the gaps were found to be very close to the expected length. In addition, there were spotty high new bone signals and patchy blood signals in the distraction gap. At the end of distraction, the mean width of the distraction gap was 3.89 ± 0.3 mm, which was close to the expected length. In the traction gap, higher echogenic bone shadows with spots and patch‐like shapes were observed, and the blood flow signal was obviously richer than those in the early stage (Fig. 4). The rats healed well in both the lengthening group and osteotomy group. After 8 weeks of mineralization, the femur was dissected and the new bone tissue was close to the normal bone tissue (Fig. 2C), and the extension rate of the left extended femur was about (5.12 ± 0.73)% compared with the opposite femur. All the fractures healed well after osteotomy in osteotomy group, and the average healing time was (7.12 ± 0.78) weeks.

### *Flow Cytometry*

**Fig. 5:**
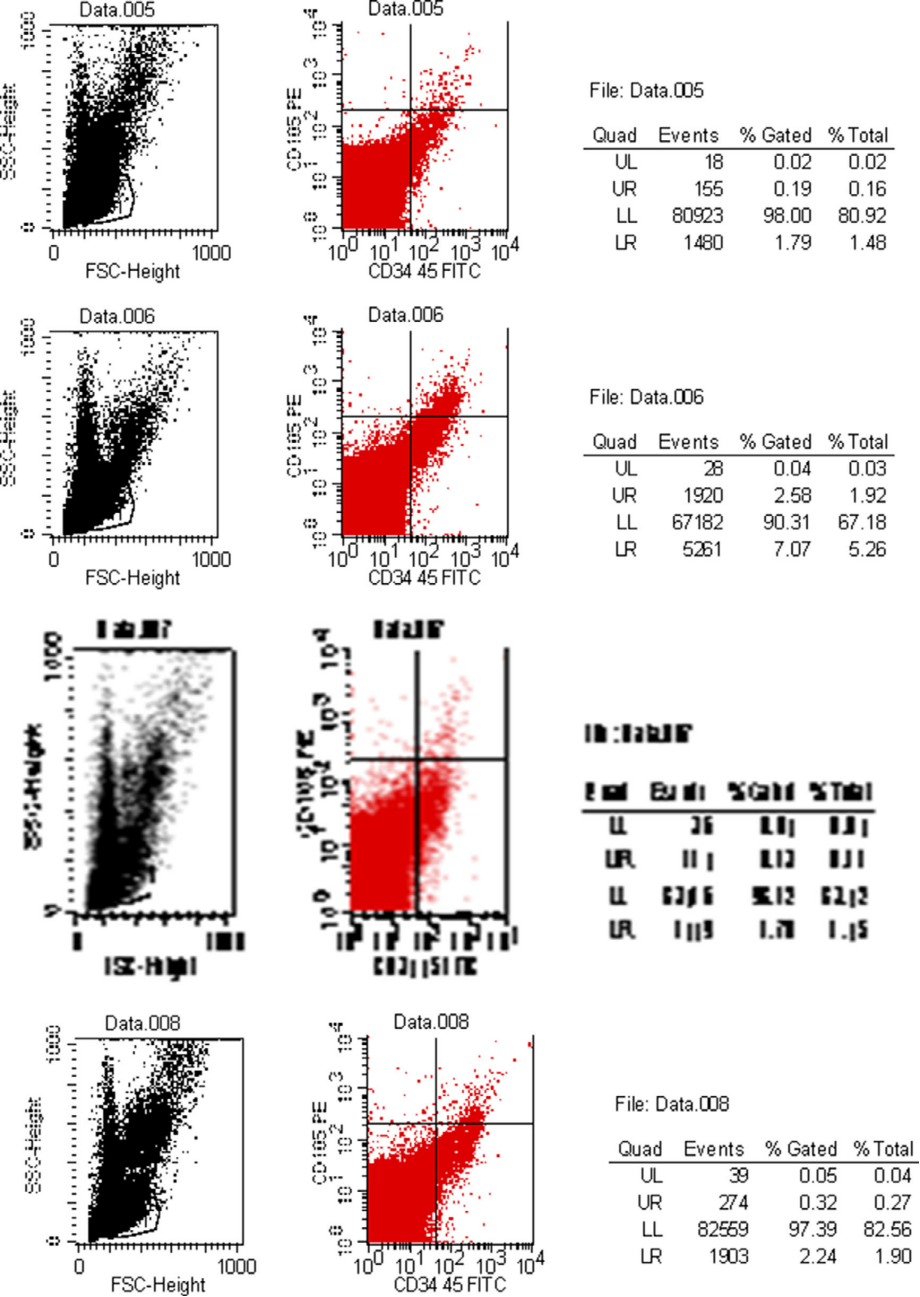
Detection of mesenchymal stem cells (MSCs) using flow cytometry. The figure shows the proportion of CD105(+), CD34, and CD45(−) MSCs in the peripheral blood of rats in lengthening group on days 1, 3, 7, and 16 of the distraction period (*n* = 6, mean ± SD, 100,000 monocytes).

In lengthening group and osteotomy group, the average concentrations of PBMSCs (3.02% ± 0.87% *vs* 2.95% ± 0.74%, respectively) were significantly increased in the early stage after osteotomy, and the average concentrations of PBMSCs on days 3, 7, and 16 after the start of traction were 5.34% ± 1.13% *vs* 3.28% ± 1.22%, 6.41% ± 1.05% *vs* 3.16% ± 0.92%, and 5.94% ± 1.23% *vs* 1.48% ± 0.52%, respectively. The concentration of PBMSCs in peripheral blood of lengthening group and osteotomy group was the same at osteotomy stage, and the difference between the two groups was no statistically significant (*P* > 0.05). After that, compared with lengthening group, the concentration of PBMSCs in osteotomy group gradually decreased and was maintained at a certain level; the difference between the two groups was statistically significant (*P* < 0.05, Table 1). Compared with osteotomy group and control group, the number of PBMSCs in lengthening group was significantly increased at each time point. The number of PBMSCs in osteotomy group was increased at the initial stage of the distraction period, but then gradually decreased. The presence of MSCs was not detected in control group (Table 1, Fig. 5).

**TABLE 1:** Percentage of peripheral blood CD105(+), CD34, and CD45(−) MSCs in the monocytes of Sprague Dawley rats at different time points during distraction osteogenesis

Groups	1 d (*n* = 6)	3 d (*n* = 6)	7 d (*n* = 6)	16 d (*n* = 6)
Lengthening group	3.02 ± 0.87	5.34 ± 1.13	6.41 ± 1.05	5.94 ± 1.23
Osteotomy group	2.95 ± 0.74	3.28 ± 1.22	3.16 ± 0.92	1.48 ± 0.52
*P* value	0.884	0.009	0.000	0.000

## Discussion

### *Establishment of* *SD* *Rat Model of Femoral Distraction Osteogenesis*

The 8‐week‐old male rats used in this experiment are stable in development, which can effectively avoid the interference of growth and development on the experimental results. The unilateral integrated external fixator used in this experiment has stable performance and convenient operation, which can meet the requirements of unilateral femoral distraction osteogenesis in rats. The weight of the micro external fixator is about 6 g, which has no significant effect on the normal activities and diet of the experimental animals. The model of SD rat femoral distraction osteogenesis established in this experiment laid a foundation for the study of the changes of circulating peripheral blood mesenchymal stem cells in distraction osteogenesis. However, in this experiment. In order to reduce the infection of the nail channel, we should pay attention to the timely replacement of the padding and strengthen the nursing of the external fixation of the nail channel during the experiment.

Due to the wide range of sources, MSCs are easy to isolate and obtain, and also easy to culture and amplify *in vitro*, making them one of the ideal seed cells for regenerative medical research, and widely used by researchers in the treatment of various diseases^11^, ^12^, ^13^. The distraction osteogenesis technique is a process of transforming mechanical stress into biochemical signals and mobilizing MSCs to migrate into the distraction gap to form bone tissue. Thus, MSCs play an important role in bone regeneration. Sunay *et al*.^10^ injected green fluorescent protein‐labeled MSCs into peripheral blood and observed that these MSCs migrated into the distraction gap in the presence of tensile stress. This finding indicates that MSCs can be recruited into the distraction gap and proliferate and differentiate into bone tissue^14^. However, whether the body's own MSCs can be mobilized and released into the bloodstream to result in an elevated concentration in the peripheral blood remains to be verified.

Numerous studies have shown that MSCs do not have specific surface markers, making their identification a perplexing issue in research. Current studies identify MSCs only based on their biological characteristics such as cell morphology and multi‐directional differentiation potential. However, in recent years, studies have found that the surface of MSCs has a high expression of CD44, CD90, and CD105 and a low expression of CD31, CD34, and CD45^15^, ^16^. Therefore, we used CD105, CD34, and CD45 to label MSCs in this study.

### *Distraction Osteogenesis Stimulated the Increase of* *PBMSCs*

Under normal physiological conditions, the proportion of MSCs in the peripheral blood is extremely low. The existence of MSCs in the peripheral blood of healthy people has been controversial. Current studies still cannot provide strong evidence to confirm this existence. The concentration of MSCs in the blood depends primarily on the proportion of mobilized MSCs in the bone marrow^17^. Mansilla *et al*.^17^ considered that the mobilization ratio of MSCs was related to the degree of injury and age. Wang *et al*.^18^ believed that MSCs can be easily obtained from the peripheral blood of rodents. However, in the present study, CD105‐positive, CD34‐negative, and CD45‐negative MSCs were not detected from the peripheral blood of rats under normal conditions. The reason may have been that the concentration of MSCs in the peripheral blood of rats was too low under normal physiological conditions, or that the flow cytometer used was not precise enough to accurately analyze the sample.

After osteotomy, the average concentrations of PBMSCs in lengthening group and osteotomy group (3.02% ± 0.87% *vs* 2.95% ± 0.74%) were significantly increased in the initial stage of distraction osteogenesis. This result suggests that the two groups have the same reaction in the early stage of the osteotomy. Hematoma is formed around osteotomy, and the inflammatory reaction may stimulate the aggregation and proliferation of mesenchymal stem cells. This result suggests that the osteotomy‐induced trauma and the inflammatory response may have caused the increased concentration of PBMSCs in the rat.

The average concentrations of PBMSCs in lengthening group and osteotomy group at days 3, 7, and 16 (5.34% ± 1.13% *vs* 3.28% ± 1.22%; 6.41% ± 1.05% *vs* 3.16% ± 0.92%; 5.94% ± 1.23% *vs* 1.48% ± 0.52%), after the start of traction were significantly increased in lengthening group after osteotomy. At the same time, the PBMSCs concentration in osteotomy group decreased gradually compared with that in osteotomy group. This suggests that distraction osteogenesis can promote the mobilization of MSCs in rats and release them into peripheral blood, and these PBMSCs will migrate to the distraction space to participate in bone regeneration. Gradually remove blood clots, necrotic soft tissue, and dead bone, make hematoma organic, and gradually form granulation tissue. At the same time, osteogenic cells, osteocytes, and bone matrix of osteotomy necrosis can release endogenous growth factors to the surrounding site and transform to osteoblasts, participating in the process of bone formation.

At the same time, the average concentration of PBMSCs in osteotomy group decreased gradually to 1.48% ± 0.52% on day 16 after osteotomy. When the pulling reaches the predetermined length, the pulling stops and then enters the fixed period. The main process of new bone formation in distraction osteogenesis is intramembrane osteogenesis. With the passage of time, the trabeculae of bone gradually grew thicker, the arrangement gradually became regular and dense, more callus was absorbed gradually under the action of osteoclasts, the medullary cavity was reopened, the braided bone was replaced by lamellar bone, and the new bone was remolded to form normal bone tissue. The results showed that the new bone tissue was close to the normal bone tissue, the bone cortex was continuous, and the osteotomy line disappeared. Compared with the contralateral normal femur, the extension rate of the femur was about (5.12 ± 0.73)% (Fig. 2B,C).

In osteotomy group, the concentration of PBMSCs increased in the early stage of fracture, mainly because of the beginning of inflammatory reaction and the formation of hematoma, which stimulated the aggregation and proliferation of mesenchymal stem cells during the inflammatory reaction. After that, because of no continuous traction stimulation, the osteotomy end gradually became stable, and the concentration of PBMSCs in peripheral blood was stable at a low level, which was statistically significant compared with the lengthening group.

These PBMSCs can migrate to the traction gap to participate in bone regeneration. Considering MSCs as the research focus, in our future studies we hope to promote targeted mobilization and migration of MSCs, to provide new treatment methods to solve the problems of poor osteogenesis and an excessively long treatment period for distraction osteogenesis.

## Conclusion

The results of this study preliminarily showed that distraction osteogenesis can promote the mobilization of MSCs in rats and release them into the peripheral blood, which elevates the number of circulating PBMSCs significantly. However, the present study also has some limitations, such as the small number of experimental animals and the lack of human trials. We will conduct human trials later to further confirm our findings.
